# A phase IV, randomized, multicenter, open-label trial comparing efficacy and systemic exposure for a standard weight-based dose versus a fixed dose of plerixafor in combination with G-CSF in patients with Non-Hodgkin’s lymphoma weighing ≤70 kg

**DOI:** 10.1038/s41409-018-0253-y

**Published:** 2018-06-12

**Authors:** John Kuruvilla, Cheng-Hwai Tzeng, Seok-Goo Cho, Seok Jin Kim, Jih-Luh Tang, Yaming Su, Jingyang Wu, Rita Vargo, Peter Cheverton

**Affiliations:** 1 Princess Margaret Cancer Centre, Toronto, Canada; 2 Taipei Veterans General Hospital, Taipei, Taiwan; 3 Catholic Medical Centre St Mary’s Hospital, Seoul, Korea; 4 Samsung Medical Centre, Seoul, Korea; 5 National Taiwan University Hospital, Taipei, Taiwan; 6 Sanofi Oncology, Cambridge, MA USA

**Keywords:** Randomized controlled trials, Haematological cancer

## Abstract

A randomized, multicenter, open-label study explored the effect of a fixed-dose (FD) of plerixafor versus the approved weight-based (WB) dose for the mobilization of hematopoietic stem cells (HSCs) in patients with non-Hodgkin’s lymphoma and a body weight of ≤70 kg. After mobilization with granulocyte colony-stimulating factor (G-CSF) 10 μg/kg/day for 4 days, patients were randomized 1:1 to either plerixafor FD 20 mg (*n* = 30) or WB 0.24 mg/kg (*n* = 31) on the evening of Day 4. Co-primary endpoints were the proportion of patients achieving ≥5 × 10^6^ CD34^+^ cells/kg in ≤4 days of apheresis, and total systemic exposure to plerixafor (area under the concentration–time curve from 0 to 10 h [AUC_0–10_]). There was no statistically significant difference between the proportion of patients attaining the primary efficacy endpoint (60% FD arm, 55% WB arm; *P* = 0.395). Exposure to plerixafor was greater in the FD arm relative to the WB arm; however, there was no appreciable difference regarding fold increases of peripheral blood CD34^+^ cells. The safety profile was similar between treatment groups. These results suggest there is no statistically significant difference in HSC mobilization with a standard WB dosing regimen of plerixafor plus G-CSF in patients with low body weight compared with an FD regimen.

## Introduction

In patients whose B-cell or T-cell non-Hodgkin’s lymphoma (NHL) is refractory, or has relapsed after first-line therapy, high-dose chemotherapy followed by autologous hematopoietic stem cell transplantation (auto-HSCT) is considered the standard of care in eligible patients [1–8]. Historically, chemotherapy and/or cytokines have been used to mobilize peripheral blood hematopoietic stem cells (HSCs) for collection by apheresis, and the CD34^+^ cell count is evaluated to determine HSC content prior to transplant. The optimum number of CD34^+^ cells for auto-HSCT is 5 × 10^6^ cells/kg of body weight, with 2 × 10^6^ CD34^+^ cells/kg being the minimum number required [5]. Studies have shown that engraftment success correlates with the number of CD34^+^ cells infused [5, 9, 10].

Plerixafor is a small molecule that selectively antagonizes chemokine receptor-4 (CXCR-4) by blocking the binding of its cognate ligand, stromal cell-derived factor-1 (SDF-1). Plerixafor was shown to increase the mobilization of CD34^+^ cells into the peripheral blood and offers a treatment option for patients undergoing stem cell mobilization for auto-HSCT. In a randomized phase II study in patients with NHL or multiple myeloma, plerixafor was well tolerated, and the combination of plerixafor and granulocyte colony-stimulating factor (G-CSF) increased the likelihood of obtaining ≥5 × 10^6^ CD34^+^ cells/kg in fewer apheresis days compared with G-CSF as a single agent [11]. Subsequently, two phase III, randomized, double-blind, placebo-controlled, multicenter studies demonstrated that the combination of plerixafor plus G-CSF was well tolerated, and mobilized significantly higher numbers of stem cells than G-CSF alone in patients with NHL or multiple myeloma [12, 13]. Plerixafor is approved in multiple regions for use in combination with G-CSF for the mobilization of HSCs in patients with NHL or multiple myeloma undergoing high-dose chemotherapy followed by auto-HSCT. In Europe, it is approved for use in such patients whose cells mobilize poorly. During the regulatory review of data from the two pivotal studies, a trend for a lower proportion of patients achieving the target of ≥5 × 10^6^ CD34^+^ cells/kg in ≤4 days of apheresis in those weighing <85 kg compared with >85 kg was noted in the NHL study (AMD3100-3101). The US Food and Drug Administration (FDA) suggested that this may be due to lower drug exposure in patients with lower body weight, and requested that this finding be explored in a controlled clinical setting. The FDA requested that a clinical study should be performed in NHL patients comparing the standard weight-based (WB) dosing regimen to a fixed dosing regimen in patients weighing ≤70 kg. Therefore, a clinical study in NHL patients to compare the standard, approved, subcutaneously (SC) administered, WB plerixafor dosing regimen of 0.24 mg/kg with a fixed-dose (FD) regimen of 20 mg/kg SC in patients with NHL weighing ≤70 kg after both sets of patients had received G-CSF for 4 days was conducted. We report here the results from this study.

## Subjects and methods

### Study design

This was a prospective, phase IV, multicenter, randomized, open-label study conducted at seven sites in four countries: Taiwan, Republic of Korea, United States, and Canada (ClinicalTrials.gov Identifier: NCT01164475). The study was conducted in compliance with the Declaration of Helsinki, Good Clinical Practice guidelines, and the laws, regulations, and applicable guidelines of the relevant countries, and was approved by each institutional review board. All patients provided written informed consent.

### Population

Eligibility criteria included: age 18–78 years; a biopsy confirmed diagnosis of NHL in first or second complete or partial remission following the first-line or second-line therapy, only where the first auto-HSCT was planned; body weight ≤70 kg (rationale for weight-based measurements described in Supplementary methods and Supplementary Table S1); an Eastern Cooperative Oncology Group (ECOG) performance status ≤1; at least 4 weeks since last cycle of cancer therapy including rituximab; white blood cell count >2.5 × 10^9^/L; absolute neutrophil count >1.5 × 10^9^/L; and platelet count >100 × 10^9^/L. Patients also had to have adequate renal, hepatic, cardiac, and pulmonary function, as determined by institutional guidelines. Exclusion criteria included: failed previous HSC collections or collection attempts; prior auto-HSCT or allogeneic HSCT; treatment with granulocyte/macrophage-CSF or pegfilgrastim within 3 weeks prior, or G-CSF within 14 days prior, to study start; chronic lymphocytic leukemia; and active central nervous system malignancy.

### Study treatment

Following screening, eligible patients received a mobilization regimen consisting of G-CSF (10 μg/kg/day) for 4 days administered by SC injection (Neupogen^®^ [Amgen Inc.] or GRAN^®^ [Kyowa Hakko Kirin Co., Ltd.] only). The baseline peripheral blood CD34+ cell count was determined on the morning of Day 4 and used to assign patients to one of two groups: <10 CD34^+^ cells/μL and ≥10 CD34^+^ cells/μL. Prior to the first dose of plerixafor on the evening of Day 4, patients were randomized in a 1:1 ratio within each CD34^+^ group to receive either plerixafor WB 0.24 mg/kg SC or FD 20 mg SC. Randomization was performed using an automated interactive voice response system incorporating a central randomization and drug supply scheme.

Plerixafor dosing was timed to allow a 10–11-h interval between dosing on Day 4 and the initiation of apheresis on Day 5. Following a dose of G-CSF 10 μg/kg SC 1 h (±15 min) prior, standard apheresis was performed using three blood volumes (±10%). The same regimen was administered (i.e., plerixafor administration in the evening and G-CSF 1 h prior to apheresis the following morning) for a maximum of four aphereses or until ≥5 × 10^6^ CD34^+^ cells/kg had been collected. The decision to continue treatment and apheresis was based on the cumulative cell yield/kg after each apheresis session.

### Endpoints

The co-primary endpoints were: (1) the proportion of patients achieving the target collection of ≥5 × 10^6^ CD34^+^ cells/kg in ≤4 days of apheresis, and (2) total systemic exposure to plerixafor.

Venous blood samples were taken on Days 4 and 5 immediately prior to G-CSF administration and then on each subsequent day of apheresis (~9–10 h post-plerixafor dosing) to evaluate the yield of peripheral blood CD34^+^ cells using fluorescence-activated cell-sorting analysis. Yields evaluated by local laboratories were used for the primary analysis reported here.

Venous blood samples for the determination of plerixafor plasma concentrations were collected prior to the first dose of plerixafor on the evening of Day 4, at 0.5, 1, and 4 h post-dose, just prior to the dose of G-CSF on Day 5, and immediately prior to the first apheresis on Day 5. Plasma concentrations were determined by validated liquid chromatography tandem mass spectrometry methods (lower limit of quantitation 5 ng/ml). Total systemic exposure to plerixafor was determined by assessment of the area under the concentration–time curve from 0 to 10 h following administration (AUC_0–10_). Estimates of other pharmacokinetic (PK) parameters included maximum observed concentration (*C*_max_), time to maximum concentration (*t*_max_), and half-life (*t*_1/2_). PK parameters were calculated using WinNonlin software (PKDMS Version 2.1 with WinNonlin Professional Version 5.2.1; Pharsight).

Secondary endpoints evaluated were the proportion of patients achieving the collection of ≥2 × 10^6^ CD34^+^ cells/kg in ≤4 days of apheresis, the number of days of apheresis required to collect ≥5 × 10^6^ and ≥2 × 10^6^ CD34^+^ cells/kg, the daily number of CD34^+^ cells/kg collected, and the total number of CD34^+^ cells/kg collected in up to four aphereses.

Safety assessments were performed throughout the study treatment period, and included reporting of treatment-emergent adverse events (AEs) up to the 24-h follow-up visit and treatment-emergent serious AEs (SAEs) up to the 30-day follow-up visit or first dose of myeloablative chemotherapy, physical examination, vital signs, and clinical laboratory evaluations.

### Statistical analysis

Based on study AMD3100-3101 (NCT00103610) [12], a sample size of 70 patients who weighed ≤70 kg (35 patients per treatment arm) would provide 80% power to detect a 30% improvement in the proportion of patients achieving the stem cell collection target of ≥5 × 10^6^ cells/kg in ≤4 days of apheresis in the FD arm, assuming that the proportion of patients in the WB arm achieving this target was 52%, at an alpha level of 0.05.

All randomized patients were included in the efficacy and safety analyses. A logistic regression model was used to compare the treatment effect in achieving the efficacy target, adjusted for country and baseline peripheral blood CD34^+^ cell count, with the WB arm as the reference. The two-sided test of significance had a Type I error rate of 0.05.

Total systemic exposure to plerixafor was determined from the PK estimate of AUC_0–10_ for each patient using a linear model that included treatment as the independent variable. For the mean test:reference ratio, the predefined equivalence bounds for the 90% confidence interval (CI) were 0.70–1.43. The relationship between treatment response and systemic exposure was explored using logistic regression models. All summaries and statistical analyses were generated using SAS Version 9.0 or higher.

## Results

### Patient characteristics

A total of 71 patients were screened and 61 patients were randomized (30 to the FD arm and 31 to the WB arm). The first patient was enrolled on October 13, 2010 and the last patient completed the study on February 26, 2013. Patient characteristics are summarized in Table 1. Due to the inclusion criterion of body weight ≤70 kg, the majority (87%) of patients randomized in this study were enrolled in Taiwan or the Republic of Korea and only 13% of patients were from the United States or Canada. Fifty-seven patients (93%) completed the study and two patients from each group were withdrawn (FD arm: lack of efficacy [*n* = 1], protocol violation [*n* = 1]; WB arm: reason missing [*n* = 1], progressive disease [*n* = 1]).

**Table 1:** Patient baseline demographic and clinical characteristics

	G-CSF + Plerixafor 20 mg SC FD (*n* = 30)	G-CSF + Plerixafor 0.24 mg/kg SC WB (*n* = 31)
Age (years), mean ± SD	46.1 ± 13.4	47.8 ± 13.6
Men/women, *n*	18/12	17/14
Baseline peripheral blood CD34^+^ cell count, n (%)		
<10 cells/μL	20 (66.7)	21 (67.7)
≥10 cells/μL	10 (33.3)	10 (32.3)
Disease type, *n* (%)		
B-cell	22 (73.3)	22 (71.0)
T or NK cell	8 (26.7)	9 (29.0)
Disease status, *n* (%)		
Complete remission	24 (80.0)	19 (61.3)
Partial remission	6 (20.0)	12 (38.7)
Remission number, *n* (%)		
First	20 (66.7)	18 (58.1)
Second	10 (33.3)	13 (41.9)
Prior therapy, *n* (%)		
Chemo/immuno-therapy	30 (100)	31 (100)
Radiation	2 (6.7)	4 (12.9)
ECOG performance status, *n* (%)		
0	26 (86.7)	21 (67.7)
1	4 (13.3)	10 (32.3)
Body weight (kg)		
Mean ± SD	61.4 ± 6.8	60.7 ± 8.9
Median (min, max)	61.0 (48.5, 70.0)	64.0 (34.2, 70.0)

*ECOG* Eastern Cooperative Oncology Group, *FD* fixed dose, *G-CSF* granulocyte colony-stimulating factor, *SC* subcutaneous, *SD* standard deviation, *WB* weight-based

### Co-primary endpoints

**Fig. 1:**
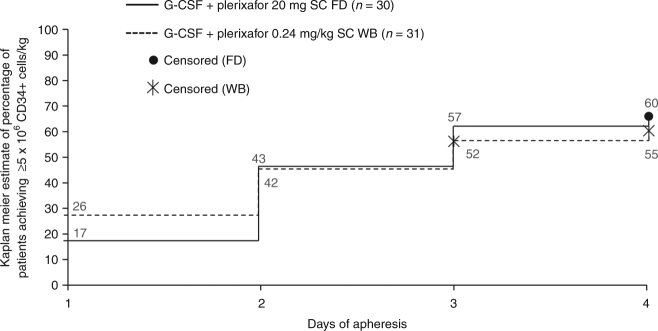
Proportion of patients reaching ≥5 × 10^6^ CD34^+^ cells in ≤4 days of apheresis. Labels on graph show percentage of patients. FD fixed dose, G-CSF granulocyte colony-stimulating factor, SC subcutaneous, WB weight-based

The co-primary efficacy endpoint of collection of ≥5 × 10^6^ CD34^+^ cells/kg was achieved by 18 of 30 (60.0%) patients in the FD group and 17 of 31 (54.8%) patients in the WB group (Fig. 1; Supplementary Table S2). The difference between dosing groups was not statistically significant (odds ratio, 1.91; 95% CI 0.44, 9.17; *P* = 0.395).

PK parameters are summarized in Table 2. The co-primary PK endpoint of relative systemic exposure by AUC_0–10_ indicated higher total systemic exposure in the FD group (Table 2). The least squares ratio of geometric means for FD versus WB AUC_0–10_ was 1.43 (90% CI 1.32, 1.54), which is outside of predefined equivalence bounds.

**Table 2:** Summary of PK parameters

	G-CSF + Plerixafor 20 mg SC FD (*n* = 30)	G-CSF + Plerixafor 0.24 mg/kg SC WB (*n* = 31)
AUC_0–10_ (ng*h/ml)		
Mean ± SD	4040 ± 602	2820 ± 436
Geometric mean (CV%)	3990 (14.9)	2790 (15.4)
*C*_max_ (ng/ml)		
Mean ± SD	957 ± 216	711 ± 136
Geometric mean (CV%)	933 (22.5)	(698) (19.1)
*T*_max_ (h)		
Median (min, max)	0.50 (0.42, 1.08)	0.50 (0.42, 1.02)

*AUC*_*0–10*_ area under the concentration–time curve from 0 to 10 h, *C*_*max*_ maximum observed concentration, *CV* coefficient of variation, *FD* fixed dose, *G-CSF* granulocyte colony-stimulating factor, *PK* pharmacokinetics, *SC* subcutaneous, *SD* standard deviation, *T*_*max*_ time to maximum concentration, *WB* weight-based

### Secondary endpoints

The globally accepted minimum target number of CD34+ cells for auto-HSCT of ≥2 × 10^6^ cells/kg was achieved in similar proportions of patients in both treatment arms: FD group, 28 of 30 patients (93.3%); WB group, 28 of 31 patients (90.3%) (Supplementary Table S1). The median time to reach the target of ≥5 × 10^6^ CD34^+^ cells/kg was 3 days in both treatment groups, and the median time to reach ≥2 × 10^6^ CD34^+^ cells/kg was 1 day in the FD group and 2 days in the WB group.

The median cumulative number of CD34^+^ cells/kg collected was comparable between the two treatment groups (5.35 × 10^6^ and 5.24 × 10^6^ for the FD and WB groups, respectively). The fold increase from baseline in peripheral blood CD34+ cells on Day 5 was similar in the FD and WB dosing groups (mean fold increases of 5.43 and 5.09, respectively) (Supplementary Table S1) despite higher plerixafor exposure in the FD cohort.

### Exploratory analyses

**Fig. 2:**
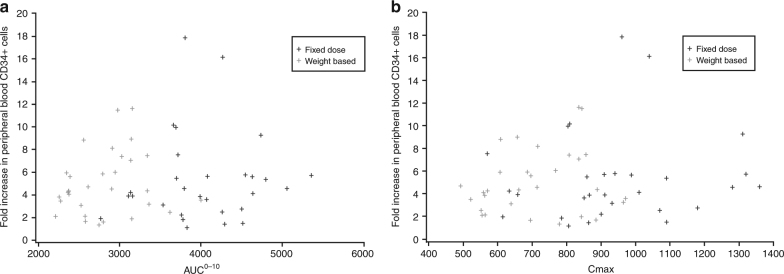
Plot of individual values for **a** AUC_0–10_ and **b** *C*_max_ versus fold increase in peripheral blood CD34^+^ cells by treatment group, where fold increase is the ratio of peripheral blood CD34^+^ level on Day 5 versus Day 4. AUC_0–10_, area under the concentration–time curve from 0 to 10 h; *C*_max_, maximum observed concentration

There was no notable relationship in this patient population between plerixafor exposure and increase in peripheral blood CD34^+^ cells (Fig. 2a, b). Logistic regression analysis confirmed that there was no statistically significant relationship between treatment response and systemic exposure after adjusting for country and baseline peripheral blood CD34^+^ cell count. In multiple logistic regression analysis, the only baseline factor with a significant relationship to response was the baseline peripheral blood CD34^+^ count on Day 4 prior to first administration of plerixafor. A subgroup analysis also indicated that a pre-apheresis count of ≥10 cells/µL was associated with a greater proportion of patients reaching ≥5 × 10^6^ CD34^+^ cells/kg in ≤4 days of apheresis compared with <10 cells/µL (100% vs. 40% in the FD arm; 100% vs. 33% in the WB arm) (Supplementary Table S1).

### Safety

The observed safety profile in this study was consistent with the known safety profile of plerixafor, and no unexpected AEs were reported. A similar number of patients in the FD and WB arms experienced AEs (87% and 84%, respectively), which were typically grade 1–2. The frequency of drug-related AEs was also similar (40% and 32%, respectively). The most common AEs (≥20% in either group) were platelet count decreased (FD 47%, WB 26%), hypokalemia (FD 17%, WB 23%), diarrhea (FD 27%, WB 13%), nausea (FD 13%, WB 23%), and anemia (FD 17%, WB 23%). The proportion of patients experiencing grade 3–4 AEs was similar in the FD and WB arms (33% and 36%, respectively); those occurring in >1 patient in either group were platelet count decreased (FD 27%, WB 19%), thrombocytopenia (FD 0%, WB 6%), and hypocalcemia (FD 7%, WB 3%). Three patients in the WB arm developed treatment-emergent SAEs (2 progressive disease and 1 cellulitis), which were considered unrelated to study drug. There were no AEs leading to discontinuation of plerixafor and no deaths during the study.

## Discussion

Plerixafor plus G-CSF is approved for stem cell mobilization prior to auto-HSCT based on two phase III, randomized, double-blind, placebo-controlled, multicenter studies, which demonstrated the safety and efficacy of this combination [12, 13]. Regulatory review of the data in the pivotal NHL study suggested that there may be slightly lower HSC mobilization in patients with low body weight in that trial, which might potentially be associated with a lower plerixafor exposure. Therefore, this randomized trial was performed to clarify if any significant differences in mobilization success rates could be identified between a 20-mg FD dose and the standard 0.24-mg/kg WB dose of plerixafor in this specific patient population. In this trial, no significant difference was identified between the FD and WB arms in the proportion of patients achieving the co-primary efficacy endpoint of an HSC collection of ≥5 × 10^6^ CD34^+^ cells/kg, or in the median time to reach this target. This was despite demonstration of a higher relative systemic exposure to plerixafor (the PK co-primary endpoint) in the FD group as determined by AUC_0–10_ and *C*_max_. The safety profile was similar in both treatment groups and consistent with the known safety profile of plerixafor.

The proportions of patients achieving the target cell collection (60.0% in the FD group and 54.8% patients in the WB group) were consistent with that seen in the pivotal phase III NHL study (59.3%) [12]. In the present study, the globally accepted minimum target number of ≥2 × 10^6^ CD34^+^ cells/kg was achieved in similar proportions of patients in both treatment arms (FD group, 93.3%; WB group, 90.3%), and was also consistent with the observed proportion (86.7%) in the pivotal study. Additional data are available from a European plerixafor compassionate use program reported by Hübel et al. [14], which enrolled 270 patients with NHL who had previously failed mobilization/collection, or who were determined to be unable to mobilize sufficient HSCs based on peripheral blood CD34+ cell counts during mobilization. The patients, ranging in weight from 43 to 132 kg (median 72 kg) and having received a median of 2 prior chemotherapy regimens, underwent HSC mobilization with plerixafor (0.24 mg/kg) and G-CSF (±chemotherapy as determined by individual investigators). The minimum target number of ≥2 × 10^6^ CD34^+^ cells/kg was achieved in 64.8% of patients (median count 2.56 × 10^6^ CD34^+^ cells/kg).

Factors such as apheresis blood volumes, activity of G-CSF, prior chemotherapy, or pre-apheresis peripheral blood CD34^+^ cell counts may have contributed to lower apheresis yields observed in the pivotal study in the low-weight NHL patients. Other studies investigating factors affecting apheresis yields have not found evidence that body weight is a contributing factor [15, 16]. A retrospective analysis of patients with NHL (*n* = 238) or multiple myeloma (*n* = 602) who underwent mobilization with chemotherapy followed by G-CSF found no effect of body weight, sex, age, or previous irradiation therapy on the incidence of poor mobilization, whereas the total number of previous chemotherapy cycles did have a significant impact [16]. In a position statement on autologous HSC mobilization by experts from the European Group for Blood and Marrow Transplantation, factors predicting poor mobilization or mobilization failure include older age, more advanced disease, prior chemotherapy (number of lines, type), and previous irradiation, with low CD34^+^ cell count in peripheral blood before apheresis being the most robust [15]. This is supported by our subgroup analysis showing that a pre-apheresis count of ≥10 cells/µL was associated with a greater proportion of patients achieving the target cell collection endpoint compared with <10 cells/µL.

The results of this prospective randomized trial align with the available data and do not identify statistically significant or clinically meaningful differences in HSC mobilization efficacy between an FD and a WB dosing regimen of plerixafor plus G-CSF in patients with low body weight, despite higher total systemic PK exposure with an FD schedule in this patient population. Furthermore, the higher PK exposure in the FD group did not translate into a clinically meaningful increase in reported AEs. The inference is that the issue seen by the assessors in the pivotal NHL study was more likely to have been related to other confounding factors or chance, and that the use of the approved plerixafor WB dose of 0.24 mg/kg does not compromise efficacy or safety in patients with a body weight of ≤70 kg.

## Electronic supplementary material

Supplementary methods

Supplementary Table S1

Supplementary Table S2
