# Supplementary material for: A phase IV, randomized, multicenter, open-label trial comparing efficacy and systemic exposure for a standard weight-based dose versus a fixed dose of plerixafor in combination with G-CSF in patients with Non-Hodgkin’s lymphoma weighing ≤70 kg
Source: Bone Marrow Transplant. 2018 Jun 12;54(2):258–64. doi: 10.1038/s41409-018-0253-y (PMC6365372; doi:10.1038/s41409-018-0253-y)
Supplement: Supplementary file 1 — Supplementary methods [file 41409_2018_253_MOESM1_ESM.docx]

**Supplementary methods**

In order for the fixed dose to be sufficiently different from the WB dose, the Marketing Authorization Holder selected a weight cut-off of <70 kg for the NHL patient population in this study. At this cut-off, the doses under investigation would be a 20-mg fixed dose versus a dose of <16.8 mg (based on a 0.24-mg/kg dose in a 70-kg patient). By design, this study would allow for adequate separation of drug exposures between the two groups (see Supplementary Table S1).
