# Supplementary material for: A phase IV, randomized, multicenter, open-label trial comparing efficacy and systemic exposure for a standard weight-based dose versus a fixed dose of plerixafor in combination with G-CSF in patients with Non-Hodgkin’s lymphoma weighing ≤70 kg
Source: Bone Marrow Transplant. 2018 Jun 12;54(2):258–64. doi: 10.1038/s41409-018-0253-y (PMC6365372; doi:10.1038/s41409-018-0253-y)
Supplement: Supplementary file 2 — Supplementary Table S1 [file 41409_2018_253_MOESM2_ESM.docx]

**Supplementary Table S1: Fixed Dose by Patient Weight**

| **Patient Weight** | **Dose at 0.24 mg/kg (mg)** | **Predicted Exposure******(ng/hr/ml)*** |
| --- | --- | --- |
| 60 kg | 14.4 | *3654* |
| 65 kg | 15.6 | 3958 |
| 70 kg | 16.8 | 4263 |
| 75 kg | 18.0 | 4567 |
| 80 kg | 19.2 | 4872 |
| 85 kg | 20.4 | 5176 |

*Values calculated for a patient with CrCL=80 mL/min
