# Supplementary material for: A phase IV, randomized, multicenter, open-label trial comparing efficacy and systemic exposure for a standard weight-based dose versus a fixed dose of plerixafor in combination with G-CSF in patients with Non-Hodgkin’s lymphoma weighing ≤70 kg
Source: Bone Marrow Transplant. 2018 Jun 12;54(2):258–64. doi: 10.1038/s41409-018-0253-y (PMC6365372; doi:10.1038/s41409-018-0253-y)
Supplement: Supplementary file 3 — Supplementary Table S2 [file 41409_2018_253_MOESM3_ESM.docx]

**Supplementary Table S2** Summary of primary, secondary, and exploratory endpoints

|  | G-CSF +  Plerixafor 20 mg SC  FD  (n = 30) | G-CSF +  Plerixafor 0.24 mg/kg SC  WB  (n = 31) |
| --- | --- | --- |
| ≥5 × 10^6^ CD34+ cells/kg in ≤4 days of apheresis^a^  Reached target, n (%)  Median time to reach target, days | 18 (60.0)  3 | 17 (54.8)  3 |
| Odds ratio (95% CI)^b^ | 1.91 (0.44, 9.17); *P* = .395 | |
| ≥2 × 10^6^ CD34+ cells/kg in ≤4 days of apheresis  Reached target, n (%)  Median time to reach target, days | 28 (93.3)  1 | 28 (90.3)  2 |
| Cumulative number of CD34+ cells/kg collected, median | 5.35 × 10^6^ | 5.24 × 10^6^ |
| Fold increase from baseline in peripheral blood CD34+ cells on Day 5, mean | 5.43 | 5.09 |
| Patients reaching ≥5 × 10^6^ CD34+ cells/kg in ≤4 days according to pre-apheresis count, n/N (%)  ≥10 cells/µL  <10 cells/µL | 10/10 (100.0)  8/20 (40.0) | 10/10 (100.0)  7/21 (33.0) |
| Supportive analyses^c^ |  |  |
| 5 × 10^6^ CD34+ cells/kg in ≤4 days of apheresis  Reached target, n (%) | 19 (63.3) | 16 (51.6) |
| Odds ratio (95% CI)^b^ | 2.05 (0.58, 7.6); *P* = .266 | |
| ≥2 × 10^6^ CD34+ cells/kg in ≤4 days of apheresis  Reached target, n (%) | 30 (100) | 29 (93.5) |

^a^Co-primary efficacy target.

^b^Reference = weight-based dose.

^c^Supportive analyses were conducted using data from central laboratory assessment of daily apheresis yields using peripheral blood samples (the primary analyses were conducted using data from local laboratory assessments).

CI, confidence interval; FD, fixed dose; G-CSF, granulocyte colony-stimulating factor; SC, subcutaneous; WB, weight-based.
